# Heat stress in livestock under tropical climates: impacts and mitigation strategies

**DOI:** 10.1007/s11250-026-04848-7

**Published:** 2026-01-27

**Authors:** Ayoola Olawole Jongbo, Leonardo Piffer de Borba, Rayssa Marcelle Martins Pereira, Qudus Opeyemi Bello, Lenara Lohana Gregoratto, Diéli Patrícia de Souza, Oluwaseesin Aderiyike Adeyeye, Frederico Márcio Corrêa Vieira

**Affiliations:** 1 Grupo de Estudos em Biometeorologia (GEBIOMET), Universidade Tecnológica Federal do Paraná (UTFPR), Campus Dois Vizinhos, Estrada para Boa Esperança, km 04, Comunidade São Cristóvão, Dois Vizinhos, Paraná 85660-000 Brazil; 2 Centro de Ciências Agrárias, Universidade Estadual do Oeste do Paraná (Unioeste), Campus Marechal Cândido Rondon, Rua Pernambuco, 1777, Marechal Cândido Rondon, Paraná 85960-000 Brazil; 3 Federal University of Technology, PMB 704, Akure, Ondo State 340110 Nigeria

**Keywords:** Production systems, Adaptation, Ruminants, Non-ruminants, Thermal tolerance

## Abstract

This review analyses the physiological and productive consequences of heat stress in tropical livestock and evaluates the mitigation strategies. Heat stress is a significant constraint in tropical production systems, where high temperatures, humidity, solar radiation, and variable wind conditions impose thermal loads that exceed animals’ capacity for heat dissipation. With global warming and more frequent heatwaves, high-yielding breeds selected for rapid growth and productivity are becoming increasingly vulnerable, resulting in significant impacts on welfare, health, and economics. Heat stress disrupts metabolic homeostasis, feed consumption, growth and reproductive efficiency. It also compromises product quality and behaviour. In dairy systems, one of the most sensitive indicators is milk yield reduction, with high-producing cows commonly experiencing losses of 10–30% under sustained thermal stress. Physiologically, heat stress induces endocrine imbalances and oxidative stress. These changes impair immune competence and productivity. These effects scale from individuals to herds, influencing food security and the sustainability of tropical livestock systems. Environmental strategies, including shading, ventilation, and evaporative cooling, reduce heat load and improve thermal comfort. Nutritional interventions aimed at enhancing antioxidant status and metabolic resilience, along with genetic selection for thermotolerance, offer additional benefits. Precision livestock farming technologies further support adaptive management through real-time monitoring and data collection, enabling informed decision-making. However, the effectiveness of these strategies depends on species-specific physiology, production intensity, and socio-economic feasibility. To address this, an integrated framework is required to guide future research and the design of cost-effective adaptation strategies in tropical livestock systems.

## Introduction

Livestock production has intensified to meet the growing global demand for animal-source foods, a trend driven by rapid population growth and increasing per capita consumption. This surge in demand has transformed livestock production systems, promoting the widespread adoption of high-yield breeds designed to maximise productivity (Ertl et al. 2015; Herrero et al. 2015; Jongbo 2020). However, under tropical conditions, this intensification, together with the reliance on metabolically demanding genotypes, exposes animals to greater and more prolonged heat loads, thereby amplifying their vulnerability to heat stress. While these advancements have substantially improved output, they have simultaneously introduced significant challenges for maintaining animal welfare and sustaining production in hot climates. Approximately 70 billion animals are raised annually worldwide to produce meat, milk, eggs, and other products, and a significant proportion of these animals are subjected to stressful conditions that often fail to meet basic welfare principles. Consequently, the drive for high productivity has inadvertently increased animals’ metabolic heat production and heightened their susceptibility to heat stress.

Global temperatures are rising at an unprecedented pace, with 2023 and 2024 recorded as the warmest years to date and monthly averages in 2024 reaching 1.54 ± 0.13 °C above pre-industrial levels (World Meteorological Organisation, 2024). This accelerating warming trend intensifies thermal pressure on livestock systems worldwide, but its impacts are particularly acute in tropical and subtropical regions where extensive and grazing-based production systems predominate (Baumgard and Rhoads 2012; Sejian et al. 2017). In these environments, animals experience prolonged exposure to high temperatures and humidity, making heat stress a major constraint on health, welfare, and productivity (Santos et al. 2021). Consequently, understanding the physiological responses that underpin heat-stress susceptibility is essential for developing effective mitigation strategies and sustaining livestock production under a rapidly warming climate.

Among livestock species, ruminants are especially vulnerable to heat stress due to their high metabolic heat production and fermentative digestion. In grazing, semi-intensive, and feedlot systems, they are continuously exposed to environmental factors—high relative humidity, solar radiation, low wind speed, and elevated temperatures—that collectively intensify thermal strain and reduce productivity. When ambient temperatures exceed the thermo-neutral zone, ruminants exhibit physiological and behavioural adjustments to dissipate excess heat, most notably a decline in voluntary feed intake, which lowers growth performance. Heat stress also alters the ruminal microbiome, disrupting the microbial balance essential for fibre digestion and nutrient absorption, and reducing feed efficiency. Additionally, it impairs ruminal motility and fermentation, decreasing volatile fatty-acid production and compromising digestive efficiency and nutrient utilisation (Sales et al. 2021). These combined effects underscore the need to understand how environmental stressors influence digestive physiology to support sustainable ruminant production under warming climates.

Heat stress in monogastric animals such as pigs and poultry triggers rapid thermoregulatory responses (elevated respiratory rate, peripheral vasodilation, and reduced feed intake and activity). However, prolonged exposure overwhelms adaptive capacity, resulting in sustained stress hormone release, immune suppression, and oxidative stress (Belhadj Slimen et al. 2016; Gonzalez-Rivas et al. 2020). This physiological burden drives a shift toward catabolism, impairing muscle development, growth performance, and carcass composition (Prates 2025a, b). More broadly, heat stress reduces metabolic efficiency, weakens immune competence, and deteriorates product quality in pigs and poultry, posing significant challenges for tropical production systems (Alam et al. 2024; Gonzalez-Rivas et al. 2020; He et al. 2021; Patra and Kar 2021; Prates 2025b, c). These combined effects underscore the need for integrated mitigation strategies to maintain productivity in the face of rising global temperatures.

The primary objective of this review is to critically and comprehensively analyse the impact of heat stress on some livestock, drawing upon existing research to provide a deeper understanding of this pressing issue. Heat stress poses a significant threat to livestock welfare, health, and productivity, particularly in tropical climates where elevated temperatures are a persistent challenge. This review aims to evaluate the current abatement techniques employed to mitigate these effects and explore potential strategies for further improvement. In addition to analysing the physiological and behavioural impacts of heat stress on livestock, this study seeks to identify gaps in existing management practices and propose innovative interventions tailored to tropical conditions. By integrating knowledge from diverse research fields, this review aims to contribute to the development of sustainable management practices that enhance livestock resilience to thermal stress. Ultimately, this review aims to provide actionable insights to support the livestock sector in mitigating the adverse effects of heat stress, promoting animal welfare, ensuring productivity, and maintaining food security in the face of rising global temperatures.

## Cattle

Among tropical livestock species, cattle are particularly vulnerable to heat stress due to their large body mass and high metabolic heat production. Effective thermal regulation is therefore essential for sustaining normal physiological functions and productivity, particularly in regions where extreme temperatures are the norm. This section examines the impacts of heat stress on calves, beef cattle, and dairy cows, highlighting their distinct physiological and metabolic responses and the resulting implications for health, growth, reproduction, and production efficiency in hot climates. Addressing these challenges is critical for protecting animal welfare and maintaining sustainable productivity as global temperatures continue to rise.

### Temperature–humidity index (THI) and physiological responses in cattle

The temperature–humidity index (THI) is a central metric for assessing the severity of heat stress across cattle categories, as it integrates thermal and hygrometric loads into a single measure of environmental strain. Empirical evidence indicates that rising THI values cause pronounced physiological, metabolic, and productivity-related responses, which vary according to age, breed, and production stage.

In calves, increasing THI induces marked thermoregulatory and endocrine responses. As reported by Kim et al. (2022), an increase in THI from 70.0 to 87.7 can cause an elevation in heart rate and rectal temperature, indicating intensified thermal strain. This can also result in heightened cortisol concentrations, signalling activation of the hypothalamic–pituitary–adrenal axis. Concurrent increases in non-esterified fatty acids (NEFA) and reductions in serum glucose at THI values above 82.92 reflect a shift toward lipid mobilisation and impaired glucose homeostasis, underscoring metabolic strain during severe heat exposure.

In beef cattle, thermal-stress thresholds depend on breed, physiological state, and prevailing climatic conditions. Heat stress may begin at ambient temperatures exceeding 27 °C (Baêta and Souza 2010; Oliveira and Knies 2019). A widely used classification indicates that THI values of 72–78 represent mild stress, 79–88 moderate stress, and 89–98 severe stress (Armstrong 1994). These thresholds support risk assessment and management planning across production systems. A comparative summary of THI thresholds and calculated values from Kim et al. (2018) and O’Brien et al. (2010) is provided in Table 1.

**Table 1:** Temperature–humidity index (THI) thresholds for evaluating heat stress in beef cattle and calves, adapted from O’brien et al. (2010) and Kim et al. (2018)

Temperature (°C)	Relative Humidity (%)	THI Value	Source
20.0	20	63.60	O’Brien et al. (2010)
22.0	60	68.61	Kim et al. (2018)
23.0	60	70.01	Kim et al. (2018)
23.0	80	71.71	Kim et al. (2018)
29.4	20	73.08	O’Brien et al. (2010)
26.0	60	74.22	Kim et al. (2018)
26.0	80	76.51	Kim et al. (2018)
30.0	60	79.84	Kim et al. (2018)
30.0	80	82.92	Kim et al. (2018)
40.0	20	83.76	O’Brien et al. (2010)
33.0	60	84.05	Kim et al. (2018)
33.0	80	87.73	Kim et al. (2018)

In dairy cattle, THI is strongly and non-linearly associated with milk yield, feed intake, and behavioural responses that underpin thermal resilience. Breed-specific thresholds illustrate contrasting heat sensitivity: Holsteins exhibit measurable declines in milk components at a THI of 46–50, whereas Jerseys maintain stable production until the THI reaches 76–80 (Lim et al. 2021). Rising THI reduces rumination time (Moretti et al. 2017) and subsequently decreases dry matter intake (DMI), restricting nutrient availability for lactation and altering nutrient partitioning. Chen et al. (2024) estimated that each unit increase in THI reduces energy-corrected milk (ECM) by approximately 3.25%, while DMI decreases by 4.13%. Field studies corroborate these patterns: milk yield losses of 4.16–14.42% have been reported once the THI surpasses 66 (Ekine-Dzive et al., 2020). Also, climate-projection models had predicted losses of 2–7.2 kg cow⁻¹ day⁻¹ in China by 2070 (Ranjitkar et al. 2020). Even heat-adapted breeds demonstrate vulnerability; for instance, Girolando cattle may experience a loss of up to 34% of their milk yield, depending on the THI and breed composition (Negri et al. 2023). At a global scale, heat stress is expected to reduce ruminant milk and meat production by approximately 9.8% of 2005 output by 2085 under high-emission scenarios (Thornton et al. 2022). Recent modelling further shows that prolonged heat stress may reduce herd-level milk yield by up to 8.6% and increase methane-emission intensity (Chen et al. 2025), emphasising the systemic nature of thermal load.

Collectively, the evidence demonstrates that THI can be a powerful integrative indicator linking environmental heat load to physiological performance in calves, beef, and dairy cattle. Its impacts on thermoregulation, endocrine balance, feed intake, and productivity highlight the necessity of early warning systems and targeted mitigation strategies to safeguard welfare and ensure sustainable production in tropical and rapidly warming climates.

### Calves

#### Long-term growth effects of heat stress on calves

Heat stress (HS) during both prenatal and postnatal stages significantly compromises calf development by impairing growth, weakening immunity, and altering nutrient metabolism. Exposure of pregnant cows to HS disrupts placental function and foetal growth, resulting in lower birth weights and reduced weaning performance (Tao et al. 2012a; Jurkovich et al. 2024). These early-life impairments have long-term consequences, as reduced weaning weight is closely associated with diminished carcass quality and overall productivity (Greenwood and Cafe 2007). Collectively, evidence indicates that heat stress affects energy and protein metabolism in calves, thereby limiting their lifetime growth potential and contributing to substantial economic losses across cattle production systems.

#### Metabolic and endocrine responses

O’Brien et al. (2010) demonstrated that Holstein calves exposed to heat stress exhibit significantly reduced plasma glucose concentrations. This hypoglycaemia is primarily associated with elevated basal insulin levels, a distinctive response compared to the lower insulin concentrations observed in pair-fed animals under thermoneutral conditions. The increase in insulin appears to be an adaptive endocrine mechanism that reallocates metabolic resources to maintain thermal homeostasis. Importantly, these metabolic alterations occur independently of feed intake, underscoring the direct influence of thermal stress on post-absorptive glucose metabolism. Similarly, Kim et al. (2022) reported reduced glucose levels in heat-stressed calves, indicative of a glucose-sparing mechanism that shifts energy dependence toward fat and protein reserves. This metabolic shift reflects an altered prioritisation of energy substrates in response to elevated thermal load.

Heat stress also alters protein metabolism, as reflected in significant reductions in circulating essential amino acids (AAs), including lysine, glutamic acid, valine, and histidine (Kim et al. 2022). These reductions are attributed to enhanced AA catabolism for gluconeogenesis and impaired gastrointestinal absorption resulting from heat-induced splanchnic vasoconstriction (Abdelnour et al. 2019). Consequently, the supply of protein precursors to muscle and adipose tissues is diminished, compromising growth and tissue development. Supporting this, Gao et al. (2017) observed elevated blood urea nitrogen (BUN) and reduced plasma-free AA levels in heat-stressed calves, indicating increased AA mobilisation for energy production. The depletion of gluconeogenic AAs plays a central role in these metabolic disturbances. However, nutritional interventions, particularly diets enriched with lysine and glutamic acid, have been shown to mitigate these effects by improving nitrogen utilisation and supporting protein synthesis under heat stress conditions (Kim et al. 2022). Collectively, these studies indicate a metabolic shift from carbohydrate toward protein catabolism under heat stress, highlighting the energetic cost of thermoregulation.

### Beef cattle

#### Heat stress in beef cattle: an emerging concern

Similar physiological disturbances also occur later in life, especially in beef cattle raised under extensive tropical conditions. Although beef cattle have received less scientific attention than dairy cattle, heat stress in these systems is becoming increasingly important. Beef breeds generally produce less metabolic heat and show greater thermotolerance than dairy breeds (St-Pierre et al. 2003). However, in tropical and subtropical regions such as Brazil, where cattle are frequently raised outdoors with limited shade and few cooling strategies, heat stress remains a persistent challenge (Giannone et al. 2023). Under these conditions, calves and cows are often exposed to prolonged periods of high temperatures, and the adoption of mitigation measures, such as sprinklers or shade structures, is still uncommon. Despite this widespread exposure, the long-term effects of heat stress on growth trajectories, carcass quality, and finishing weights remain poorly documented and require further investigation.

#### Impacts of heat stress on physiology, metabolism, and meat quality

Heat stress prior to slaughter disrupts the standard post-mortem conversion of muscle energy, producing measurable declines in meat quality. Acute exposure elevates core temperature and accelerates glycogen breakdown, resulting in excess lactic acid and a rapid pH decline, which leads to pale, soft, exudative (PSE) meat with a low water-holding capacity (Bejaoui et al. 2023; Mia et al. 2023). In contrast, chronic heat exposure reduces feed intake. It depletes muscle glycogen reserves, limiting lactic acid formation and leading to dark, firm, dry (DFD) meat with a higher ultimate pH, an outcome more common in ruminants exposed to prolonged thermal stress (Adzitey and Nurul 2011; Mia et al. 2023). Elevated temperatures further intensify protein and lipid degradation, contributing to poorer texture, colour stability, and shorter shelf life.

To clarify these patterns, Table 2 summarises the key characteristics of PSE and DFD meat, including pH thresholds and associated thermal conditions.

**Table 2:** Summary of PSE and DFD meat characteristics, pH thresholds, and associated thermal conditions

Parameter	PSE meat	DFD meat	References
Typical pH pattern	Rapid post-mortem decline; ultimate pH < 5.8	Slow decline; ultimate pH > 6.2	Bejaoui et al. 2023; Adzitey and Nurul 2011; Mia et al. 2023
Colour characteristics	Pale, soft, exudative	Dark, firm, dry	Bejaoui et al. 2023; Mia et al. 2023
Water-holding capacity (WHC)	Low WHC due to protein denaturation	High WHC due to limited protein denaturation	Adzitey and Nurul 2011; Mia et al. 2023
Muscle glycogen status	Accelerated glycogen breakdown; excess lactic acid	Low glycogen reserves; limited lactic acid	Mia et al. 2023; Adzitey and Nurul 2011
Thermal conditions	Acute heat stress	Chronic, prolonged heat exposure	Bejaoui et al. 2023; Mia et al. 2023
Primary physiological mechanism	Rapid glycolysis leads to a rapid pH drop	Glycogen depletion leads to insufficient lactic acid	Adzitey and Nurul 2011
Species most affected	Poultry, pigs, cattle	Ruminants	Bejaoui et al. 2023; Adzitey and Nurul 2011

Physiological and metabolic alterations that occur during heat stress further compound these impairments in carcass quality. In beef calves, O’Brien et al. (2010) reported that heat stress reduced dry matter intake (DMI) by 12%, causing average daily gain (ADG) to fall from 1.24 kg/day to − 0.09 kg/day. Water intake more than doubled in heat-stressed animals, reflecting the need for evaporative cooling. Plasma glucose fell by 7%, while insulin rose by 33%, indicating a shift in nutrient partitioning towards thermoregulation rather than growth. Plasma urea nitrogen (PUN) increased by 77%, demonstrating intensified protein catabolism, and non-esterified fatty acids (NEFA) remained unchanged, suggesting that lipolysis was not the primary compensatory mechanism.

Heat stress also modifies pre-slaughter physiology in ways that directly influence meat traits. Moderate positive correlations between blood glucose and lactate, observed by Hultgren et al. (2022), indicate consistent activation of the hypothalamic–pituitary–adrenal (HPA) axis. Strong correlations among colour traits demonstrate that stress-induced biochemical shifts influence myoglobin oxidation. Shorter transport and reduced handling in mobile abattoirs partially mitigated these responses, improving water-holding capacity relative to stationary abattoirs.

Heat-driven panting accelerates glycogen depletion (Nielsen et al. 2022; Burns et al. 2019), reducing the substrate required for normal post-mortem glycolysis and increasing the likelihood of DFD meat (Burns et al. 2019; Ribeiro et al. 2022). Elevated temperatures denature muscle proteins, thereby lowering their structural integrity and water-holding capacity (Khalid et al. 2023). Conversely, reduced energy intake suppresses intramuscular fat deposition, which in turn diminishes marbling (Fouad and El-Senousey 2014). Heat-induced cortisol elevation exacerbates protein and fat catabolism (Moura et al. 2021), and dehydration impairs muscle function and exudate control (Mia et al. 2023). Oxidative stress further accelerates lipid and protein oxidation, leading to off-flavours and reduced shelf life (Chauhan et al. 2023; Gonzalez-Rivas et al. 2020; Mia et al. 2023).

Collectively, these physiological, metabolic, and biochemical disturbances form a unified heat-stress syndrome that undermines both productivity and meat quality. Reduced feed intake, altered glucose–insulin regulation, and heightened protein catabolism decrease feed efficiency and promote liveweight loss. Glycogen depletion compromises normal post-mortem metabolism, increasing the risk of DFD meat, while acute hyperthermia predisposes animals to PSE conditions. Thermal and oxidative damage to muscle proteins and lipids further deteriorates tenderness, juiciness, water-holding capacity, and shelf life. These combined process alterations translate into measurable economic losses in tropical beef systems, emphasising the importance of adaptive management practices.

#### Developmental effects of prenatal and postnatal heat stress

Heat stress impacts cattle throughout the production cycle, beginning in the uterus. Cows exposed to thermal stress during gestation give birth to calves with lower birth weights due to maternal hyperthermia and impaired nutrient delivery (Seyed Almoosavi et al. 2021; Wang et al. 2020). These effects persist postnatally, reducing growth potential and impairing muscle development. During the calf and weaning stages, heat stress reduces feed intake and shifts metabolic energy away from growth, resulting in slower weight gain, delayed musculoskeletal development, and ultimately smaller carcasses (Greenwood and Cafe 2007).

### Dairy cattle

#### Impact of heat stress on dairy cattle performance and physiology

Heat stress is a major constraint in dairy production, compromising animal welfare, productivity, and milk quality. It arises when metabolic heat production exceeds the animal’s capacity to dissipate it, leading to thermal imbalance and triggering a cascade of physiological, endocrine, and metabolic disruptions (Khan et al. 2023). These disturbances impair immune function, suppress reproductive performance, and reduce overall productivity (Collier et al. 2017). Among the earliest and most economically relevant indicators is a decline in milk yield, with losses of 10% to 25% commonly reported during periods of intense heat (West 2003). Such reductions carry substantial economic costs; for example, heat stress is estimated to cause annual losses of approximately USD 900 million to the U.S. dairy sector alone (Baumgard and Rhoads 2012; Khan et al. 2023).

#### Alterations in milk composition under heat stress

Milk-fat and milk-protein concentrations are highly sensitive to thermal load. Collier et al. (2017) reported that milk-fat content decreases by approximately 0.4% during heat-stress events, while milk-protein content declines by about 0.2%, even during cooler seasons. Under more severe conditions, these reductions are amplified. Habiba et al. (2025) documented decreases of 14.6% in milk fat and 17.3% in milk protein at THI 80. Rhoads et al. (2009) similarly observed protein losses up to 2% depending on management conditions. Importantly, protein reductions may occur independently of fat reductions, indicating different metabolic pathways affected by heat stress (Cowley et al. 2015; Fabris et al. 2017, 2019). Boonkum et al. (2024) reported a negative correlation of − 0.256 between THI and milk-protein concentration, and a − 0.48 correlation between THI and the fat-to-protein ratio. Environmental exposure also influences milk composition. Hill and Wall (2014) found that extended sunshine hours in outdoor systems reduce milk-fat content relative to indoor housing, underscoring the role of environment–housing interactions in modulating heat-stress impacts.

#### Milk quality, udder health, and mineral balance

Heat stress compromises not only macronutrient composition but also the nutritional, hygienic, and processing qualities of milk. Casein levels decline, while urea content increases, reducing the suitability of the milk for cheese manufacture and other dairy-processing applications (Amenu et al. 2004). Elevated somatic cell count (SCC), a consequence of immunosuppression during heat stress, increases susceptibility to mastitis and intramammary infections (Ludovico et al. 2015; Sandrucci et al. 2014). Nonetheless, hygiene practices such as pre-dipping and environmental sanitation can substantially mitigate SCC elevation (Beaupied et al. 2022; Igono et al. 1988; Sandrucci et al. 2014; Wegner et al. 1976). Thermal load also disrupts mineral balance. Some indigenous breeds show reduced milk calcium during hot periods, while Tharparkar cattle exhibit significant potassium loss due to excessive sweating (Maurya et al. 2020). These observations highlight the importance of targeted electrolyte supplementation during prolonged heat exposure to maintain homeostasis and milk quality.

## Sheep

Having examined the physiological, metabolic, and carcass-related consequences of heat stress in cattle, it is important to contextualise these responses within a broader ruminant framework. Although cattle exhibit marked reductions in feed efficiency, growth, and meat quality under thermal strain due to their lower heat-dissipation capacity, sheep display a comparatively higher thermotolerance. This interspecific contrast justifies shifting the focus to sheep, a species whose adaptive traits offer an alternative model for understanding resilience to elevated temperatures.

This section, therefore, examines the impact of heat stress on sheep performance, detailing how elevated temperatures influence their physiological, productive, and reproductive efficiency. Sheep possess a well-developed capacity to cope with climatic variability, enabling them to produce meat, wool, milk, and leather efficiently. Within their thermal comfort zone, they rely predominantly on sensible heat exchange pathways to maintain thermal equilibrium at a minimal energetic cost (Kadzere et al. 2002; Polsky and von Keyserlingk 2017).

When exposed to high ambient temperatures, sheep initially mount acute thermoregulatory responses, shifting from sensible to evaporative heat loss mechanisms such as panting and sweating, which increase respiratory muscular effort and energy expenditure (Vieira et al. 2021). These immediate responses are accompanied by peripheral vasodilation, which enhances heat dissipation but reduces blood flow to internal organs, altering nutrient absorption and overall metabolic efficiency (Al-Dawood 2017).

With sustained heat exposure, sheep activate a suite of adaptive physiological and endocrine adjustments aimed at re-establishing homeostasis. These include increases in heart and respiratory rates (Santos et al. 2021) and alterations in hormonal profiles, notably elevated glucocorticoids and reductions in thyroid hormones and growth hormones. These collectively suppress metabolic rate and growth potential (AL-Ramamneh 2023). Feed intake declines while water consumption rises, reducing nutrient availability and digestive efficiency and ultimately impairing growth and productivity (Bernabucci 2012; Kadzere et al. 2002). Heat exposure also accelerates glycogen mobilisation, leaving muscle stores depleted at slaughter and compromising carcass characteristics and meat quality (Mia et al. 2023).

Heat stress additionally exerts profound effects on reproductive function. Elevated glucocorticoids and suppressed anabolic hormones disrupt the hypothalamic–pituitary–gonadal (HPG) axis, leading to reduced gonadotropin release, impaired follicular development, decreased conception rates, and compromised offspring viability (Macías-Cruz et al. 2013; Marai et al. 2007; Nóbrega et al. 2011). This endocrine–reproductive disruption, combined with declining nutritional status, directly lowers flock fertility and overall production efficiency.

Collectively, these acute and adaptive responses demonstrate how heat stress impairs sheep performance across metabolic, productive, and reproductive domains, underscoring the need for targeted mitigation strategies.

### Thermoregulation and heat stress

Thermal regulation in sheep is a complex process controlled by both the endocrine and central nervous systems (Polli et al. 2020). The peripheral nervous system plays a crucial role in detecting temperature fluctuations through specialised receptors located within the skin surface. These receptors transmit thermal information to the hypothalamus, which then orchestrates a series of physiological, circulatory, and behavioural responses aimed at maintaining body homeostasis (Szolcsányi 2015). Through the mechanisms of thermogenesis (heat production) and thermolysis (heat loss), sheep strive to maintain an optimal body temperature range of 38.3 to 39.9 °C. However, this range can vary depending on factors such as environmental temperature, activities, breed, sex, age, and production category (Reece and Rowe 2017).

Under normal conditions, sheep primarily rely on sensitive heat exchange mechanisms that do not require additional energy expenditure. These include conduction, where heat is transferred through direct contact with surfaces, convection, which involves heat exchange with surrounding fluids such as air, and radiation, where long-wave infrared radiation is emitted to regulate body temperature (Bohmanova et al. 2007; Kadzere et al. 2002). However, these mechanisms become insufficient when exposed to temperatures outside their thermoneutral zone, typically between 20 and 30 °C (Revell 2016). To compensate, sheep initiate physiological and behavioural adaptations that, while helping restore homeostasis, result in increased energy expenditure, ultimately reducing productive and reproductive efficiency (McManus et al. 2022).

In thermally challenging environments, sheep transition from using energy-efficient sensitive heat exchange mechanisms to more demanding latent mechanisms, primarily involving evaporative cooling through respiratory and cutaneous processes. The effectiveness of these mechanisms varies, with respiratory evaporation playing a dominant role, especially in wool-covered animals, while cutaneous evaporation is less significant. Additional physiological responses include peripheral vasodilation to facilitate heat dissipation, as well as an increase in heart rate to maintain blood circulation and respiratory rate to enhance evaporative cooling. Although these responses are essential for survival, they impose a high metabolic cost, further impairing animal performance. The efficiency of evaporative cooling is heavily influenced by relative humidity (RH). For optimal thermoregulation, an RH of approximately 65% is recommended for sheep (Eustáquio Filho et al. 2011). When humidity levels exceed this threshold, evaporative cooling becomes less effective due to reduced water loss from the respiratory tract and skin, leading to increased heat retention and heightened stress levels (Barnes et al. 2004; Oke et al. 2021).

Physiological indicators such as respiratory and heart rates serve as valuable measures of thermal stress in sheep. Under normal conditions, respiratory rates range from 20 to 38 breaths per minute (Armengol et al. 2017), while resting heart rates typically fall between 65 and 80 beats per minute (Konold and Bone 2011). However, these values can fluctuate based on breed, production stage, environmental conditions, and overall health status (Armengol et al. 2017; Rashamol et al. 2018; Tüfekci and Sejian 2023; Wanjala et al. 2023). Significant deviations from these norms indicate heightened thermal stress, making these parameters useful for monitoring animal welfare and identifying necessary interventions (Armengol et al. 2017; McManus et al. 2020).

### Nutrition, productive performance, and carcass quality

Heat stress profoundly impacts the feeding behaviour and overall metabolism of sheep, triggering a cascade of physiological and biochemical changes that compromise their health and productivity. One of the most immediate responses to high temperatures is a reduction in dry matter intake (Habeeb et al. 2023; Hill and Wall 2017). While this adaptation helps minimise metabolic heat production, it comes at a significant cost, reduced nutrient absorption and energy intake. To compensate for the loss of feed intake, heat-stressed sheep consume more water (McManus et al. 2022), which increases the volume of the digestive tract. However, this adjustment accelerates the rate of passage of digesta, further hindering proper digestion and nutrient assimilation (Van Weyenberg et al. 2006; Warner 1981). Over time, these disruptions lead to depleted fat reserves, lower carcass yields, and, in severe cases, a negative energy balance (Al-Dawood 2017; Marai et al. 2007), making it difficult for the animal to maintain optimal growth and production levels. To regulate body temperature, sheep activate latent heat dissipation mechanisms such as evaporative cooling, which demands high energy expenditure. This increased energy demand, coupled with reduced nutrient intake, leads to a sharp decline in productive performance. At a physiological level, heat stress suppresses thyroid hormones such as Triiodothyronine (T3) and Thyroxine (T4) (AL-Ramamneh 2023), which play a vital role in metabolism. Similarly, growth-related hormones decline (Farooq et al. 2010), slowing overall development. At the same time, the adrenal glands respond by increasing the secretion of cortisol, a stress hormone that promotes gluconeogenesis, the breakdown of muscle glycogen to produce energy (Al-Dawood 2017). Unfortunately, this rapid depletion of muscle glycogen reserves has serious consequences for meat quality. As glycogen is essential for postmortem glycolysis, its reduction leads to lower lactic acid production, resulting in higher ultimate pH levels in the meat (Zhang et al. 2020). This change affects both the texture and appearance of the final product. Meat from heat-stressed sheep often takes on undesirable characteristics, becoming dark, firm, and dry (DFD), less tender, more prone to spoilage, and with a significantly shorter shelf life (Mia et al. 2023; Zhang et al. 2020). These deteriorations not only impact organoleptic properties such as flavour, texture, and colour but also lower the economic value of the meat (Al-Dawood 2017; Zhang et al. 2020), posing a major economic challenge for sheep farmers.

## Pigs

This section examines the impact of heat stress on pigs, a monogastric species whose thermolabile physiology contrasts markedly with that of ruminants. Pigs possess an extremely limited capacity for evaporative cooling due to their sparse sweat glands and low surface area–to–mass ratio, making them particularly susceptible to hyperthermia in tropical and subtropical environments. Consequently, heat stress disrupts key physiological processes, including nutrient metabolism, endocrine regulation, growth, reproduction, and lactation, resulting in measurable reductions in performance and overall productivity.

### Thermal sensitivity and thermoregulation in pigs

Heat stress presents a critical challenge in pig production systems due to the species’ unique physiological limitations in coping with elevated ambient temperatures. Unlike other livestock, pigs possess only rudimentary sweat glands, rendering evaporative cooling through sweating virtually ineffective (Katiyar et al. 2025). Instead, they rely on alternative mechanisms such as increased respiration and behavioural modifications, seeking shade, wallowing, and reducing feed intake, to regulate body temperature. However, these compensatory behaviours are often insufficient under persistent or extreme heat conditions, particularly in warm climates.

The pigs’ thermoneutral zone (TNZ) generally falls between 16 and 25 °C, with optimal relative humidity ranging from 60 to 80% (Arulmozhi et al. 2021; Machado et al. 2016). Within this environmental range, pigs can efficiently convert feed into productive outputs such as meat, milk, or reproductive performance. Deviation from the TNZ, especially into higher temperature ranges, results in physiological stress that disrupts homeostasis and impairs productive functions. In subtropical and tropical zones, where indoor housing conditions are closely influenced by fluctuating and elevated outdoor climates, maintaining the TNZ becomes particularly challenging.

### Impact of heat stress on growth and nutrient utilisation

Elevated temperatures impose a substantial metabolic burden on pigs by diverting nutrients and energy away from growth and reproduction toward thermoregulation. Under heat stress, pigs typically reduce their feed intake by approximately 15–20%, resulting in growth depressions of up to 21–30% and significant declines in feed conversion efficiency (Serviento et al. 2020; Romo-Valdez et al. 2022; Ross et al. 2015). At the same time, maintenance energy requirements increase as evaporative and respiratory heat-loss mechanisms intensify, creating a pronounced negative energy balance.

Heat stress also disrupts endocrine and inflammatory pathways, impairing digestion, nutrient absorption, and metabolic partitioning. These alterations shift energy utilisation away from anabolic processes and toward homeostatic maintenance, reducing protein deposition and overall growth potential. In fast-growing commercial genotypes, such metabolic strain contributes to elevated morbidity and mortality, especially under intensive production conditions (Arulmozhi et al. 2021). Collectively, these effects highlight how heat stress reconfigures nutrient and energy allocation, leading to substantial productivity and economic losses in pig systems operating in warm climates.

### Reproductive consequences of heat stress in boars and sows

The reproductive system is particularly vulnerable to thermal stress, with distinct effects observed in male and female pigs. In boars, elevated temperatures have been shown to inhibit spermatogenesis, leading to reduced semen volume, decreased sperm motility, and increased morphological abnormalities in spermatozoa (Kunavongkrit et al. 2005). Heat-sensitive boars exhibit more severe reproductive impairment and may fail to recover normal fertility levels even after thermal conditions improve. Additionally, fluctuating temperatures exacerbate this stress, making thermal adaptation even more difficult for sensitive animals (Kunavongkrit et al. 2005).

Sows are similarly affected by elevated thermal loads. Heat stress impairs hormonal regulation by reducing the secretion of gonadotropins such as luteinising hormone (LH), which disrupts ovulation and impairs follicular development. This leads to longer intervals between weaning and estrus, higher rates of anestrus, and reduced litter sizes (Katiyar et al. 2025). Thermal stress also compromises mammary gland function, which may reduce milk production and impair piglet survival and growth during lactation. These disruptions cumulatively degrade sow productivity and longevity within breeding herds.

Although ventilation and climate control systems offer partial mitigation, effective protection of swine welfare requires integrated strategies combining genetics, housing design, and nutrition to reduce heat load under increasingly variable climatic conditions.

## Poultry

Poultry are particularly vulnerable to heat stress due to their high metabolic heat production, the absence of sweat glands, and insulating plumage, which makes thermal dissipation inefficient in tropical and subtropical environments. This vulnerability has significant economic implications, as poultry supplies a substantial proportion of the world’s animal protein. Even short periods of heat exposure can markedly reduce growth, feed efficiency, and egg production (Fairchild 2017; Saeed et al. 2019; Mnisi et al. 2024).

### Effects of heat stress on growth performance

Heat stress imposes a multifactorial constraint on poultry growth, primarily by disrupting metabolic, endocrine, and nutritional processes essential for efficient tissue accretion. Elevated temperatures depress the basal metabolic rate, especially under chronic exposure, which reduces production efficiency and compromises meat quality in broilers (Akbarian et al. 2016; Brugaletta et al. 2022). This decline is driven in part by reduced secretion of thyroid hormones, particularly triiodothyronine (T3), which lowers protein synthesis, increases protein catabolism, and contributes to metabolic acidosis (Vasilatos-Younken et al. 2000). Concurrently, heat stress elevates circulating glucocorticoids, stimulating gluconeogenesis and altering body composition, thereby compromising carcass quality (Caballero 2023).

One of the earliest measurable consequences is a reduction in feed intake, as birds voluntarily limit diet-induced thermogenesis in response to high ambient temperatures (Kumar et al. 2021; Nawab et al. 2018). In tropical conditions, feed intake may fall by 5–20%, and broiler growth rate can decline by 20–40% (Kpomasse et al. 2021; Osti et al. 2017; Sesay 2022; Syafwan et al. 2011). El Melki et al. (2024) demonstrated that approximately *67%* of heat-induced growth depression is attributable to reduced intake, with the remainder arising from direct metabolic impairments, including altered gut integrity and decreased nutrient absorption.

Feed conversion efficiency also deteriorates under heat stress, as a larger fraction of metabolizable energy is redirected toward thermoregulation rather than muscle deposition (Khan et al. 2011). This inefficiency is especially evident in modern broiler strains, whose inherently high metabolic heat production heightens susceptibility to thermal load (Onagbesan et al. 2023). Taken together, these endocrine shifts, reductions in nutrient intake, and increased energy demands for maintaining thermal balance explain the substantial decline in growth performance observed in heat-stressed poultry.

The severity of growth impairment due to heat stress is influenced by a range of factors, including the intensity and duration of heat exposure, relative humidity, radiant heat load, the bird’s age and genetic background, and its thermoregulatory efficiency (Onagbesan et al. 2023). These variables modulate the physiological response to thermal stress, which means that even under similar ambient conditions, the degree of growth suppression can vary significantly. Consequently, understanding these complex interactions is essential for designing effective heat mitigation strategies to sustain poultry productivity in hot climates.

### Physiological effects of heat stress on reproduction

Heat stress induces marked physiological and endocrine disruptions that impair reproductive function in laying hens. As temperatures rise above the thermoneutral range, hens experience reduced feed intake, impaired nutrient assimilation, and hormonal imbalances that directly affect ovulation and follicular development (He et al. 2018; Hester 2017; Jongbo 2020). Elevated corticosterone suppresses gonadotropin secretion, disrupts ovarian cyclicity, and decreases follicular recruitment. Cellular damage within the ovary is exacerbated by heat-induced activation of apoptotic pathways, including FasL/Fas and TNF-α signalling, which reduces the pool of viable follicles (Li et al. 2020). Panting-induced respiratory alkalosis decreases blood CO₂ and free calcium availability, impairing the endocrine–mineral balance required for normal ovarian and shell gland function (Hester 2017; Soliman and Safwat 2020). The timing of thermal exposure also modulates these effects: daytime heat stress mainly affects shell formation, whereas nighttime exposure more strongly depresses ovulation and overall egg production (Wolfenson et al. 1979). Importantly, reproductive impairments may persist even after temperatures return to normal, indicating long-lasting disruption of ovarian physiology (Li et al. 2020; Wolfenson et al. 1979). These reproductive and productive declines represent a major constraint for sustainable and welfare-oriented poultry production in tropical and subtropical regions.

### Productive effects on egg output and egg quality

Productively, heat stress causes substantial reductions in egg number, size, and structural quality. Field studies report declines in egg production ranging from 15 to 40% (Al Amaz and Mishra 2024; Nawaz et al. 2021), while severe acute heat exposure may reduce lay by up to 11% (Kim and Lee 2023). Reduced nutrient intake under heat load limits calcium and energy availability, producing smaller eggs with thinner and weaker shells (Bekele 2021; Cornescu et al. 2023; Kim and Lee 2023). Internal quality indicators such as Haugh units also deteriorate under thermal challenge (Kim and Lee 2023). Heat stress increases the frequency of defective eggs, including soft-shelled, speckled, and misshapen eggs, owing to impaired shell gland function and reduced mineral deposition (Zhu et al. 2015). Water intake may rise to 2.5 times the normal levels, yet even brief interruptions in water supply can abruptly halt egg laying (Yohannes and Tekle 2018). Panting-related shifts in acid–base balance further compromise shell integrity, while sustained metabolic strain leads to weight loss and lower persistency of lay (Jefferson 2023). Nutritional and management interventions such as increasing dietary electrolyte balance to 360 mEq/kg (Nobakht et al. 2006) and supplementing herbs, probiotics, or botanical extracts have demonstrated potential to partially restore production under thermal stress (Ma et al. 2005; Zhu et al. 2015). Collectively, heat-induced endocrine disruption, impaired calcium and nutrient metabolism, and ovarian dysfunction translate into significant losses in egg quantity and quality.

### Effect of heat stress on health

Heat stress poses a major health concern in poultry, occurring when the equilibrium between the bird’s internal heat production and its ability to dissipate heat into the environment is disturbed. This imbalance induces a cascade of physiological and biochemical responses that compromise birds’ overall health, productivity, and welfare. One of the primary consequences of heat stress is the induction of oxidative stress, which results from the excessive generation of reactive oxygen species (ROS), and a concurrent depletion of the body’s antioxidant defences (Hu et al. 2019; Nawaz et al. 2021). This redox imbalance can damage lipids, proteins, and DNA, ultimately impairing cellular integrity and contributing to systemic dysfunction (Brugaletta et al. 2022).

Another significant physiological impact of heat stress in poultry is the alteration of acid–base balance and suppression of immune function. Chronic exposure to high temperatures has been associated with reduced lymphocyte counts, decreased immunoglobulin levels, and a weakened immune response, rendering birds more susceptible to infectious diseases (Nawaz et al. 2021; Wasti et al. 2020). Broilers under heat stress frequently exhibit immunological disorders, including lymphoid organ atrophy and diminished immunoglobulin synthesis (Aguanta et al. 2018; Ahmad et al. 2022; Liu et al. 2025). These immunosuppressive effects are often exacerbated by the overproduction of pro-inflammatory cytokines such as tumour necrosis factor-alpha (TNF-α) and interleukin-1 beta (IL-1β), which further impair immune function and intestinal barrier integrity (Nawaz et al. 2021).

The gastrointestinal tract is particularly vulnerable to the effects of heat stress. This system is central to nutrient absorption and immune surveillance, and its integrity is crucial for overall poultry health. Heat stress disrupts gut homeostasis by impairing blood flow, causing intestinal ischemia and hypoxia, and reducing gut motility and nutrient utilisation (Brugaletta et al. 2022). It also compromises the epithelial barrier by damaging tight junction proteins, leading to increased intestinal permeability, a condition often described as “leaky gut.” As a result, harmful luminal substances, including bacteria and toxins, can translocate into the bloodstream, promoting systemic inflammation and further health deterioration (Brugaletta et al. 2022; Nagpal and Yadav 2017). Heat stress is also known to affect the gut microbiome by decreasing the diversity and stability of intestinal microorganisms (Deng et al. 2012; Rostagno 2020). Alterations in the microbiota composition interfere with digestive efficiency and immune modulation, compounding the adverse effects on health and growth performance. Additionally, inflammation of the gastrointestinal tract under heat stress conditions directly affects feed intake, growth rates, and meat and egg quality (Saeed et al. 2019).

At the metabolic level, heat stress significantly alters lipid metabolism. In chickens, it promotes hepatic fat accumulation and enhances gene expression in de novo lipogenesis (Nawaz et al. 2021). Simultaneously, appetite-regulating hormones such as leptin and adiponectin are upregulated, contributing to reduced voluntary feed intake despite increased water consumption. The decline in nutrient intake under thermal stress conditions severely hampers energy balance and growth potential. Furthermore, the effects of heat stress extend to the reproductive system. In female poultry, it disrupts the secretion of reproductive hormones, impairing ovulation and egg production, while in males, it leads to a decline in semen quality and fertility (Bekele 2021). These reproductive impairments are compounded by systemic stress and endocrine disruption, which alter the function of the hypothalamic–pituitary–gonadal axis.

Thus, heat stress has a multifaceted impact on poultry health, affecting oxidative balance, immune competence, gastrointestinal function, metabolism, and reproductive performance. These physiological disruptions contribute to decreased productivity, increased morbidity, and heightened vulnerability to environmental and pathogenic challenges. Understanding these health-related consequences is essential for developing effective mitigation strategies to improve poultry resilience in hot climates.

## Mitigation strategies and future perspectives

Heat stress remains one of the most persistent barriers to sustainable livestock production, as it compromises productivity, welfare, and product quality across species. With projected increases in global temperatures, mitigation will depend on strategies that simultaneously improve the thermal environment, enhance physiological resilience, and promote long-term genetic adaptation (Most and Yates 2021; Prates 2025b; Renaudeau et al. 2012; Slayi et al. 2024). This section synthesises the main environmental, nutritional, genetic and technological interventions, emphasising their mechanisms of action, applicability under tropical conditions, and implications for future livestock systems.

### Cooling systems and environmental management

Environmental interventions remain the first line of defence against heat stress. Their effectiveness depends on species-specific physiology, production systems, and climatic conditions.

#### Ruminants

In both grazing and confined systems, ruminants experience thermal loads resulting from high ambient temperatures, solar radiation, and humidity. Shade - natural or artificial - reduces radiant heat load and surface temperature, lowering respiratory rate, rectal temperature, and behavioural indicators of stress (Améndola et al. 2016; Santos et al. 2021; Vieira et al. 2021). In confinement, combining shade with mechanical ventilation and water sprinkling enhances convective and evaporative cooling, increases feed intake, and improves milk yield (Berman 2008; Silveira et al. 2026; Strickland et al. 1989). These strategies are particularly crucial during late gestation and early lactation, when thermal stress has long-lasting effects on milk production and calf development (Jordan 2003; Roth 2022; Tao et al. 2012b). Heat-stress indices such as THI and BGHI, despite limitations, remain practical tools for guiding environmental interventions in tropical regions (Aurambout et al. 2024; Bohmanova et al. 2007; Pezzopane et al. 2019).

#### Poultry

Broilers and laying hens exhibit limited thermoregulatory capacity, making ventilation, evaporative cooling pads, and automated climate management essential (Oluwagbenga and Fraley 2023). Increasing airflow turbulence disrupts the boundary layer surrounding birds, enhancing sensible heat loss and reducing mortality (Jongbo 2018). Water sprinkling or misting further improves evaporative cooling, reducing body temperature and PSE meat incidence, an important quality concern during transport and pre-slaughter handling (Lesiów and Xiong 2024; Liang et al. 2020).

#### Pigs

Pigs’ limited sweating ability and high metabolic heat production demand cooling strategies centred on ventilation, exhaust systems, and intermittent misting (Renaudeau et al. 2006; Silveira et al. 2026). These methods effectively reduce core temperature, support feed intake and reproductive performance, and lower mortality risk during heat waves (Vitali et al. 2021). Where feasible, wallows or cooled flooring promote natural thermoregulatory behaviour and enhance comfort in semi-intensive systems.

#### Emerging and integrated cooling technologies

New technologies, including indirect evaporative systems with regenerative heat exchangers, improve thermal comfort without increasing indoor humidity, making them increasingly relevant for tropical climates (Schauberger et al. 2019, 2020). Precision environmental control, integrating sensors, fans, sprinklers, and automated decision algorithms, further strengthens climate resilience. However, adoption outside poultry and dairy remains limited due to cost and infrastructural constraints (Ferreira et al. 2024).

### Genetic strategies

Genetic approaches complement environmental interventions by enhancing inherent thermotolerance. Heat-adapted breeds often display lower production potential than temperate breeds, reflecting physiological trade-offs that must be considered in breeding programmes (Michael et al. 2022).

Two lines of action predominate:

i. **Selection and crossbreeding for adaptive phenotypes**
  Traits such as hair-coat characteristics (“slick” genotype) and reduced feathering (“Na” naked-neck gene) improve heat dissipation in cattle and poultry, respectively (Fathi et al. 2022; Pozzebon et al. 2024). These adaptations improve thermoregulation without compromising welfare but require careful assessment of correlated traits and epigenetic consequences (Oluwagbenga and Fraley 2023).
ii. **Genomic identification of thermotolerance markers**
  Advances in single-nucleotide polymorphism (SNP) arrays, targeted sequencing, and whole-genome sequencing enable high-resolution detection of alleles associated with heat resilience (Dikmen et al. 2013; Li et al. 2011; Oliveira et al. 2024; Vijayakumar et al. 2022). Despite promising results, implementation remains constrained by cost and technical capacity.

Integrating genomic tools into breeding schemes will progressively strengthen herd resilience, particularly when aligned with environmental and nutritional interventions.

### Nutritional strategies

Nutritional interventions mitigate heat stress by stabilising cellular function, supporting metabolic efficiency, and reducing endogenous heat production. These strategies can be grouped by mechanism of action:

i. **Antioxidants**.

Heat stress increases oxidative damage, making antioxidant supplementation, —vitamin E, selenium, polyphenols—effective for protecting cellular integrity, improving immune responses, and enhancing meat quality (Chauhan et al. 2021; Prates 2025a, b; Zheng et al. 2022).

ii. **Osmolytes and membrane stabilisers**.

Compounds such as betaine and taurine support osmotic balance, membrane stability, and water retention. They are particularly effective in monogastrics but also benefit ruminants exposed to high temperatures (Prates 2025a, b).

iii. **Diet reformulation**.

Increasing dietary energy density through concentrates or protected fats compensates for reduced feed intake, while optimising the protein: energy ratio minimises heat increment (Beede and Collier 1986; Conte et al. 2018; Min et al. 2019). Maintaining adequate levels of electrolytes and heat-sensitive vitamins supports acid–base balance and metabolic adaptation (Reddy et al. 2023).

iv. **Functional additives**.

Probiotics, yeast, prebiotics, plant extracts and phytochemicals enhance gut health, modulate inflammation, and improve thermotolerance (Chauhan et al. 2021; Kotsampasi et al. 2024; Sammad et al. 2020).

v. **Water management**.

Continuous access to cool, clean water is fundamental for thermoregulation and productivity. Water temperature significantly influences heat dissipation and feed intake (Conte et al. 2018; Reddy et al. 2023).

Integrated application of these nutritional tools offers the strongest protection, particularly when tailored to species, production type, and seasonal risk.

### Precision livestock farming (PLF)

PLF enhances heat-stress mitigation by coupling real-time physiological and behavioural monitoring with automated environmental control. IoT-enabled sensors, ruminal boluses, accelerometers, and thermal cameras allow continuous measurement of body temperature, respiration, activity, and microclimatic conditions (Islam et al. 2021; Ji et al. 2020; Levit et al. 2021; Nguyen and Nguyen 2023; Xiong et al. 2025).

Machine-learning models further refine management by predicting thermal stress before clinical signs appear, enabling automated adjustment of ventilation, sprinklers, or shading systems (Mylostyvyi et al. 2024). Although adoption is currently concentrated in poultry and dairy operations, expansion into other sectors is crucial for climate-resilient production.

### Future perspectives

A forward-looking strategy must extend beyond single interventions and emphasise integrated, scalable, and species-specific solutions. Three research priorities emerge:

i. **Genomics and phenomics for thermotolerance**
  Incorporating genomic markers, epigenetic signatures, and high-resolution phenotyping into breeding programmes is essential for developing long-term resilience.
ii. **AI-driven environmental management**
  Combining PLF technologies with predictive climate models will allow proactive adjustment of cooling systems, reducing mortality and production losses during extreme weather events.
iii. **Sustainable applications for small-scale and tropical systems**
  Low-cost cooling technologies, locally adapted diets, and regionally appropriate breeds are critical for resilience in smallholder systems where climate impacts are most severe.

Together, these mitigation and future-oriented strategies represent a coherent framework for improving resilience, sustaining productivity, and enhancing welfare in livestock systems facing intensifying thermal challenges.

## Conclusion

Heat stress is a major constraint across livestock species, particularly in tropical climates where high temperature and humidity overwhelm thermoregulatory capacity. Despite physiological differences among species, the core impacts are similar. This includes disrupted metabolic function, reduced productivity, impaired reproduction, and compromised health and welfare, highlighting heat stress as a common biological and economic limitation to livestock systems.

Effective mitigation requires combining environmental, nutritional, and genetic strategies. Cooling systems, shade, and improved ventilation must be integrated with targeted dietary interventions and long-term selection for heat-tolerant animals. These approaches are most successful when adapted to local conditions and embedded within broader management practices.

Future priorities include developing cost-effective cooling solutions for tropical farms, advancing genetic and molecular tools for heat tolerance, and expanding precision monitoring technologies to detect early physiological stress. Strengthening research, innovation, and technology transfer will be essential to enhance resilience and sustain animal production under accelerating climate change.

## Data Availability

No new data were generated or analysed in the development of this review. Therefore, data sharing is not applicable.

## Declarations

The authors declare that no funds, grants, or other support were received during the preparation of this manuscript.
